# Streamlining Borrelia burgdorferi cultivation using quantitative PCR screening

**DOI:** 10.1099/jmm.0.002123

**Published:** 2026-02-06

**Authors:** Beat M. Greiter, Semjon Sidorov, Ester Osuna, Annina Schalch, Lisa M. Greiter, Elena Robinson, Michelle Seiler, Michelle Bressan, Frank Imkamp, Oliver S. Beer, Leslie Ens, Oliver Nolte, Adrian Egli, Christoph Berger, Patrick M. Meyer Sauteur

**Affiliations:** 1 Division of Infectious Diseases and Hospital Epidemiology, Children’s Research Center, University Children’s Hospital Zurich, University of Zurich, Zurich, Switzerland; 2 Emergency Department, University Children’s Hospital Zurich, Zurich, Switzerland; 3 Institute of Medical Microbiology, University of Zurich, Zurich, Switzerland

**Keywords:** *Borrelia burgdorferi*, culture, Lyme disease, phase-contrast microscopy, spirochete

## Abstract

**Introduction.** Direct detection of *Borrelia burgdorferi* by culture is considered the gold standard for confirming Lyme disease (LD). However, *B. burgdorferi* culture is not routinely used in clinical practice or research due to its lengthy protocol and low success rate. This study aimed to streamline the process by integrating a specific quantitative PCR (qPCR) screening early into the *B. burgdorferi* culture workflow for identification of cultures that are likely to yield viable spirochetes.

**Methods.** Thirty-two blood plasma and 11 cerebrospinal fluid (CSF) samples were collected from 32 children with serologically confirmed LD and incubated in modified Kelly-Pettenkofer medium for up to 9 weeks, with weekly assessments for viable spirochetes using microscopy. After 3 weeks, the presence of *B. burgdorferi* DNA in culture was assessed by qPCR targeting the *B. burgdorferi flagellin B* gene. The estimated copy number of the target template was compared to the assay’s 95% limit of detection (LOD).

**Results.** After 9 weeks of incubation, viable spirochetes were observed in 2 (*n*=2/32, 6.3%) plasma cultures and 3 (*n*=3/11, 27.3%) CSF cultures. These were only observed in cultures showing copy numbers above 95% LOD in qPCR testing at week 3 (*n*=2/3 plasma cultures, 66.7%; *n*=3/3 CSF cultures, 100.0%).

**Conclusion.** Culturing *B. burgdorferi* is challenging and, despite a high workload, often not successful. qPCR may serve as an effective screening tool for *B. burgdorferi* cultures, enabling the culturing process to be streamlined by prioritizing cultures with target copy numbers exceeding the 95% LOD of the qPCR assay.

## Introduction

*Borrelia burgdorferi sensu lato* is the causative agent of Lyme disease (LD), the most prevalent tick-borne illness in the Northern Hemisphere [1]. The most common manifestations of LD in children include erythema migrans (EM; early localized infection), Lyme neuroborreliosis (LNB; early disseminated infection) and Lyme arthritis (LA; early or late disseminated infection) [2].

To date, the gold standard for diagnosing LD is considered to be the direct detection of *B. burgdorferi* by culture [3], but it involves labour-intensive and lengthy protocols. *B. burgdorferi* has a long *in vitro* doubling time (~12 h) and is sensitive to high and low temperatures, high oxygen levels and freshness of culture media components. It has to be cultured in specialized and rich culture media like the Barbour-Stoenner-Kelly or the modified Kelly-Pettenkofer (MKP) medium under microaerophilic conditions at 33–37 °C for up to 9–12 weeks [4,6]. Further, clinical specimens have a very low bacterial load (0.1 culturable bacteria per millilitre of blood) [7], resulting in a low culture success rate even with large culture inoculation volume. Reported sensitivity depends on the origin of clinical samples and ranges from 1.5 to 44% for blood [7,14] and from 3 to 30% for cerebrospinal fluid (CSF) [8,9, 11, 12, 15,19]. Therefore, despite its diagnostic value, culturing *B. burgdorferi* is challenging and is neither routinely performed in clinical settings nor commonly used in research.

Improving the culturing procedure is essential for advancing research and diagnostics into clinical isolates, epidemiology, virulence factors and host-pathogen interactions, which would deepen our understanding of *B. burgdorferi* biology and LD pathogenesis. We hypothesize that one approach to improving the procedure is to reduce the workload associated with *B. burgdorferi* culture. This could be achieved by minimizing the number of negative cultures maintained for up to 9 weeks through early screening for *B. burgdorferi* DNA using a quantitative PCR (qPCR) assay. Several PCR assays targeting different *B. burgdorferi* genes exist, but detection rates in blood and CSF of LD patients are relatively low (5–30%) [3,8]. Studies on other pathogens have shown that pre-culturing original samples can improve PCR detection rates, particularly when target agents are present at very low levels [20,21].

The aim of this study was to integrate specific qPCR screening into the *B. burgdorferi* culture workflow to streamline the process and reduce the maintenance workload.

## Methods

### Ethics statement

The samples used in this study were obtained from patients participating in the BRILLIANT study (ClinicalTrials.gov ID: NCT06045416), a single-centre observational cohort study involving children with suspected LD at the University Children’s Hospital Zurich. The protocol for this study was approved by the ethics committee of the Canton of Zurich, Switzerland (no. 2023-00528). Written informed consent was obtained from all parents and from children from 14 years of age.

### Sample collection and procedure

Whole blood and CSF samples from 32 paediatric patients diagnosed with LD were collected at the Children’s Hospital Zurich between 1 April 2024 and 31 March 2025. The diagnosis of LD was clinically and serologically confirmed according to current guidelines [22]. LD manifestations included LNB (*n*=13), EM (*n*=12) and LA (*n*=7). CSF was collected as part of routine diagnostic procedures from LNB patients only; CSF was not available for two LNB patients. None of the patients were treated with antibiotics prior to blood and CSF collection.

Whole blood and CSF samples were kept at room temperature (RT) until further processing and culture inoculation, which was performed within 18 h after sampling. Whole blood samples were centrifuged at 1,000 g for 5 min to separate plasma for culture inoculation, while CSF samples were used directly for culture inoculation.

### *B. burgdorferi* culture

MKP cultivation medium was used for *B. burgdorferi* culture and prepared according to published protocols [4]. After preparation, basic MKP medium was stored at −20 °C for up to 3 months at the Institute of Medical Microbiology, University of Zurich, Switzerland. Complete MKP medium was prepared by supplementing basic MKP medium with 6% rabbit serum, 7% gelatin and 35% BSA (Supplementary protocol, pages 5–6, available in the online Supplementary Material). Complete MKP medium was stored at 4 °C and used within 1 month.

For inoculation of clinical samples, 1 ml of plasma or CSF was mixed at a ratio of 1 : 5 with pre-warmed complete MKP medium in 10 ml glass tubes with attached lids allowing gas exchange. Liquid cultures were incubated at 37 °C up to 9 weeks under microaerophilic conditions using a sealed anaerobic chamber (GenBox 7L, bioMérieux, Marcy-l’Étoile, France) and atmosphere generators (bioMérieux, Marcy-l’Étoile, France). To maintain cultures, the medium was refreshed every 3 weeks by centrifuging the culture at 4,000 g for 10 min, discarding the supernatant and resuspending the pellet in fresh pre-warmed complete MKP medium. Cultures were monitored weekly by loading 10 µl of liquid culture onto a disposable haemocytometer (Bioswisstec, Schaffhausen, Switzerland) and searching for viable spirochetes using phase-contrast microscopy at 400× magnification.

Prior to the experiment, the culture method was tested using the *B. burgdorferi* B31 and V149 laboratory strains (obtained from the Leibniz Institute DSMZ-German Collection of Microorganisms and Cell Cultures GmbH, Braunschweig, Germany). After 3 weeks of incubation without changing culture medium, we observed a plateau with a later decrease in bacterial concentration (by microscopic examination, data not shown). Therefore, week 3 was selected as the PCR testing timepoint. Throughout the study, a laboratory strain was grown as an independent positive control alongside clinical cultures under the same conditions.

### Subculture, storage and reactivation of *B. burgdorferi* cultures

After 3 weeks of incubation, two 1 ml aliquots of each liquid culture were collected and washed twice in 0.85% NaCl buffer by centrifugation at 10,000 g for 10 min. Washed pellets were resuspended in 0.5 ml of 0.85% NaCl for later qPCR assay and in 1 ml of 10% glycerol for long-term storage, respectively. Both aliquots were frozen at −80 °C.

During 9 weeks of incubation, cultures in which viable spirochetes were detected by microscopy were sub-cultured until reaching a bacterial concentration of 1×10^8^ per millilitre (as determined by microscopy) and subsequently frozen in 1 ml of 10% glycerol at −80 °C for long-term storage. If no viable spirochetes were detected, the culture was discarded.

Frozen cultures were re-activated by thawing 1 ml of frozen glycerol stock at RT and inoculating at a ratio of 1 : 5 with pre-warmed complete MKP medium. Cultures were maintained and monitored in the same manner as freshly inoculated cultures, as described above. We could re-activate frozen cultures from all culture-positive donors frozen at 1×10^8^ bacteria per millilitre.

### *B. burgdorferi*-specific duplex qPCR

As an internal extraction and PCR inhibition control, samples were spiked with phocine herpesvirus 1 (PhHV) culture (obtained from the European Virus Archive–GLOBAL, Marseille, France; reference no.: 011 V-00884). A duplex qPCR assay targeting *flagellin B* (*flaB*) gene was used to detect *B. burgdorferi* DNA and PhHV genome to serve as a control. Primer/probe sequences used in the PCR assay have been described previously [23, 24]. Primers and probes were synthesized by Microsynth (Balgach, Switzerland), as detailed in Table S1.

### Plasmid standards

To quantify *B. burgdorferi* DNA copy numbers, a standard for the *flaB* qPCR assay was generated by cloning *flaB* qPCR target sequence (174 bp, as detailed in Table S1) into a proprietary plasmid backbone (TIB Molbiol Syntheselabor GmbH, Berlin, Germany). A seven-point dilution series (10^8^ to 10^1^ copies per microlitre) diluted in Tris-EDTA buffer was prepared, and standards were run in triplicates on every qPCR plate.

### *B. burgdorferi* DNA isolation

Frozen bacterial samples corresponding to 0.4 ml of culture were used. Culture aliquots were thawed on ice, centrifuged at 10,000 g for 10 min, resuspended in 190 µl of PBS and then spiked with 10 µl of a 1 : 1,000 diluted PhHV internal control. DNA isolation was performed using the Quick-DNA Miniprep Plus Kit (D4069, Zymo Research Corporation, Irvine, CA, USA), according to the manufacturer’s instructions. DNA was eluted in 50 µl of DNA elution buffer.

### qPCR reaction

The qPCR reaction was pipetted on a 96-well plate on ice, with the following components: 10 µl of TaqMan^™^ Fast Advanced Master Mix (Applied Biosystems, Thermo Fisher Scientific, Waltham, MA, USA), 5 µl of primer/probe mix (final concentrations of 0.9 nM for primers and 0.25 nM for probes) and 5 µl of sample DNA, resulting in a total reaction volume of 20 µl. The qPCR reaction conditions included an initial incubation at 50 °C for 2 min, followed by polymerase activation at 95 °C for 20 s, 45 cycles of denaturing at 95 °C for 1 s and annealing/extension at 60 °C for 20 s.

The qPCR assay, including DNA isolation, was performed in three individual batches. For no template controls (NTCs), we used DNase-free water instead of sample DNA. NTC was included on every qPCR plate for a combination of two primers/probe pairs (targeting *flaB* and PhHV). The qPCR reactions were run on a CFX96^™^ Real-Time System with the C1000 Touch Thermal Cycler (Bio-Rad Laboratories, Hercules, CA, USA).

The qPCR results were analysed using the CFX Maestro^™^ software 2017 (Bio-Rad Laboratories, Hercules, CA, USA), and the baseline threshold for the quantification cycle (Cq) values was set above the background noise within the exponential phase of the amplification curve.

We generated standard curves for each experiment using Microsoft Excel 2019 software (Microsoft Corporation, Redmond, WA, USA), applying a linear regression model to the mean Cq values of the plasmid standard triplicates. We then calculated the estimated number of *B. burgdorferi* target templates per qPCR reaction. These copy numbers were then normalized to 1 ml of the tested culture sample, followed by logarithmic transformation (log of quantity). The slopes and R squares of the standard curves from the three individual experiments were as follows: −3.846 and 0.997, −3.943 and 0.998 and −4.027 and 0.998, respectively.

### Estimation of limit of detection (95% limit of detection)

Quantitative sensitivity of the *B. burgdorferi*-specific qPCR assay was determined using a well-growing *B. burgdorferi* culture (4.19×10^6^ *B. burgdorferi* DNA copies per millilitre at harvest, measured by qPCR) established from a patient sample. Six replicates of a serial dilution (6×1 : 10 dilutions, ranging from 4.19×10^5^ to 4.19×10^0^ *B. burgdorferi* DNA copies per millilitre) were prepared from the same culture, resulting in a total of 36 samples.

DNA extraction and qPCR reaction were performed separately for each sample, as described above. Based on these results, the cut-off value (95% limit of detection [LOD]) was estimated as the lowest number of target sequence (estimated analytes/bacterial cells) per 1 ml of *B. burgdorferi* suspension that could be reliably detected by the qPCR assay in 95% of the replicates. The 95% LOD was estimated using the R software environment, analysing the mean log of quantity per 1 ml of dilutions and the percentage of qPCR-positive replicates using a generalized linear model with a logit link function, as recommended by Burns and Valdivia [25]. The 95% LOD was determined to be 6.38×10^3^ copies or 3.80 log of quantity of target template per 1 ml of *B. burgdorferi* culture (Fig. S1).

### Microscopy

Microscopy and image documentation were performed using a Leica DM IL LED Fluo microscope with a Leica K3C colour camera.

To capture images of *B. burgdorferi* spirochetes at the first detection timepoint, 1 ml aliquot of culture containing viable spirochetes was used for slide preparation as follows: the washed cell pellet was resuspended in 0.2 ml of a 3:1 mixture of methanol (99.9%) and acetic acid (99%). Then, 10 µl of the bacterial suspension was placed on a glass slide and gently spread by holding the slide at a 60° angle. After air-drying at RT, the slide was washed sequentially in 80 and 100% ethanol for 1 min each. The fixed bacterial cells were then stained by adding 10 µl of DAPI staining solution (0.125 µg ml^−1^) and covered with a coverslip. Images were done with phase-contrast and fluorescent microscopy at 400× magnification.

### Statistical analysis

To assess differences in *B. burgdorferi* DNA copy numbers between cultures with and without detectable spirochetes by microscopy, we performed a Wilcoxon rank-sum test using the R software environment (version 4.4.0).

## Results

The qPCR testing results for *B. burgdorferi* DNA after 3 weeks and microscopy findings over 9 weeks of incubation for 32 plasma and 11 CSF cultures are shown in Table 1. NTCs included on each PCR plate yielded negative results, and the internal extraction control was successfully amplified in all tested samples.

**Table 1.** Results of *B. burgdorferi*-specific qPCR after 3 weeks and weekly microscopy over 9 weeks of cultivation of 32 patients with LD

Patient no.	Diagnosis	Plasma				CSF			
		qPCR 3 **weeks**		Microscopy 9 **weeks**		qPCR 3 **weeks**		Microscopy 9 **weeks**	
		Copies per millilitre*,†	Interpretation‡	Spirochetes first detected (day)§	Interpretation	Copies per millilitre*,†	Interpretation‡	Spirochetes first detected (day)§	Interpretation
1	LNB	583	Positive (<LOD)	na	Negative	8,087,120	**Positive (>LOD)**	33	**Positive**
2	LNB	ns	Negative	na	Negative	3,625,751	**Positive (>LOD)**	13	**Positive**
3	LNB	ns	Negative	na	Negative	511,927	**Positive (>LOD)**	56	**Positive**
4	LNB	164	Positive (<LOD)	na	Negative	1,333	Positive (<LOD)	na	Negative
5	LNB	36,527	**Positive (>LOD)**	na	Negative	840	Positive (<LOD)	na	Negative
6	LNB	ns	Negative	na	Negative	361	Positive (<LOD)	na	Negative
7	LNB	ns	Negative	na	Negative	94	Positive (<LOD)	na	Negative
8	LNB	487	Positive (<LOD)	na	Negative	ns	Negative	na	Negative
9	LNB	ns	Negative	na	Negative	ns	Negative	na	Negative
10	LNB	281	Positive (<LOD)	na	Negative	ns	Negative	na	Negative
11	LNB	493	Positive (<LOD)	na	Negative	ns	Negative	na	Negative
12	LNB	ns	Negative	na	Negative				
13	LNB	125	Positive (<LOD)	na	Negative				
14	EM	577,042	**Positive (>LOD)**	17	**Positive**				
15	EM	4,083	Positive (<LOD)	na	Negative				
16	EM	417	Positive (<LOD)	na	Negative				
17	EM	101	Positive (<LOD)	na	Negative				
18	EM	ns	Negative	na	Negative				
19	EM	ns	Negative	na	Negative				
20	EM	ns	Negative	na	Negative				
21	EM	ns	Negative	na	Negative				
22	EM	ns	Negative	na	Negative				
23	EM	ns	Negative	na	Negative				
24	EM	ns	Negative	na	Negative				
25	EM	ns	Negative	na	Negative				
26	LA	3,855,875	**Positive (>LOD)**	40	**Positive**				
27	LA	725	Positive (<LOD)	na	Negative				
28	LA	517	Positive (<LOD)	na	Negative				
29	LA	200	Positive (<LOD)	na	Negative				
30	LA	ns	Negative	na	Negative				
31	LA	ns	Negative	na	Negative				
32	LA	ns	Negative	na	Negative				

*Estimated copies of target template in 1 ml of culture.

†NS (no signal) – no amplification signal detected by qPCR above baseline threshold.

‡Interpretation of qPCR testing: negative – no *B. burgdorferi* DNA detected; positive (<LOD) – *B. burgdorferi* DNA detected, but signal is below 95% LOD; positive (>LOD) – *B. burgdorferi* DNA detected and signal above 95% LOD. 95% LOD: 6.38×103 copies per millilitre or 3.80 log(10) of quantity per millilitre (Fig. S1).

§Day after incubation start when living spirochetes were first detected by phase-contrast microscopy at 400× magnification.

CSF, cerebrospinal fluid; EM, erythema migrans; LA, Lyme arthritis; LNB, Lyme neuroborreliosis; LOD, limit of detection; NA, not available; NS, no signal.

Following a 3-week incubation period, qPCR testing revealed the presence of *B. burgdorferi* DNA in 15 (46.9%) plasma cultures (LNB, *n*=7; EM, *n*=4; and LA, *n*=4) and in 7 (63.6%) CSF cultures (LNB, *n*=7). The estimated copy numbers of the target template per 1 ml of culture sample were above the 95% LOD (6.38×10^3^ copies) in 3 (9.4%) plasma cultures (LNB, *n*=1; EM, *n*=1; and LA, *n*=1) and in 3 (27.3%) CSF cultures (LNB, *n*=3). In two of those cultures (1 plasma and 1 CSF culture), viable spirochetes were detected by phase-contrast microscopy during 3 weeks of incubation.

**Fig. 1.**
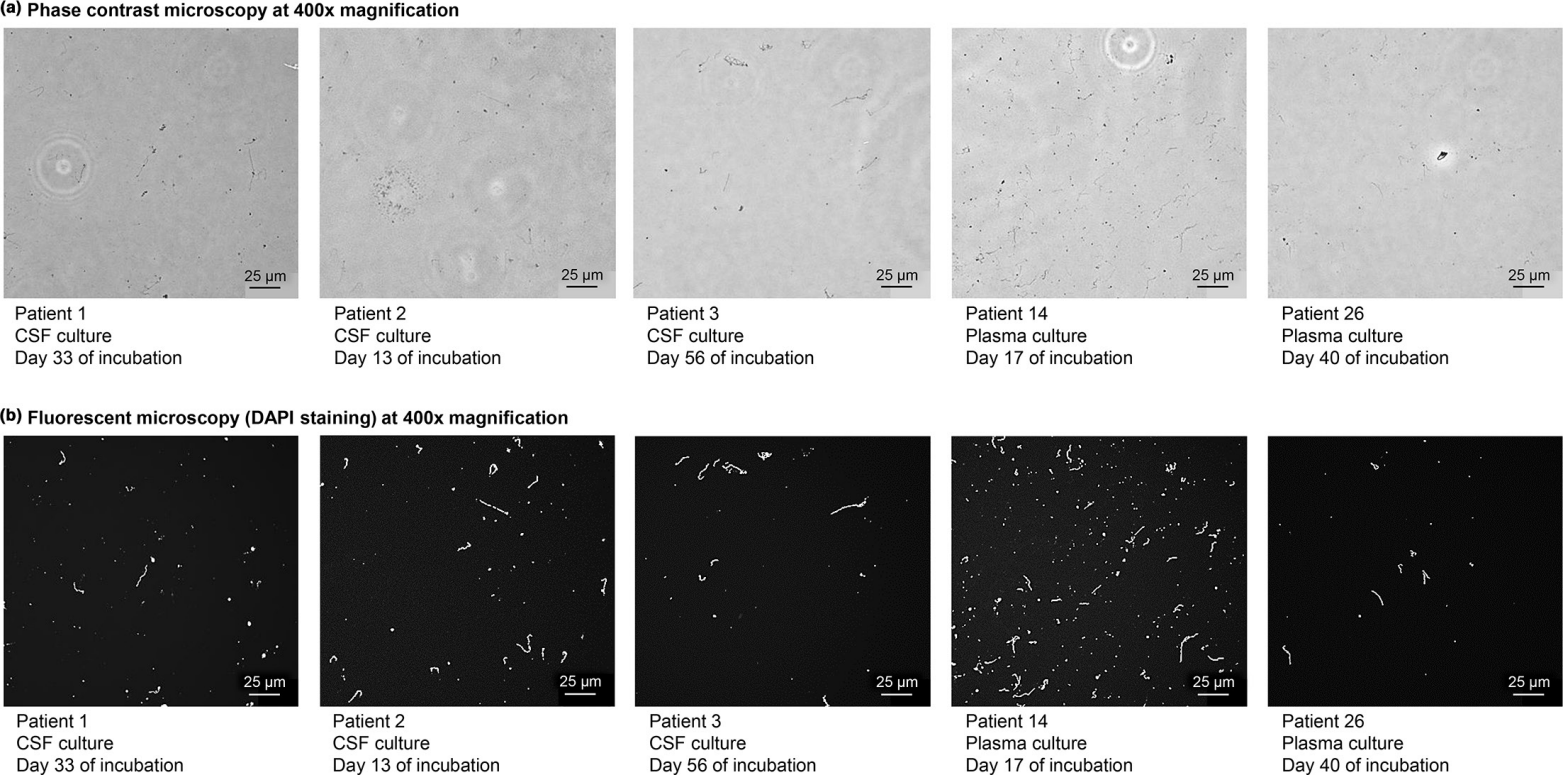
Images of *B. burgdorferi* culture isolated from blood plasma and CSF of five children with LD. Images were taken at the first timepoint (day of incubation) when viable spirochetes were detected. Abbreviation: CSF, cerebrospinal fluid.

Within 9 weeks of incubation, viable spirochetes were observed by phase-contrast microscopy in 2 (6.3%) plasma cultures (EM, *n*=1; and LA, *n*=1) and 3 (27.3%) CSF cultures (LNB, *n*=3) (Fig. 1). Spirochetes were only observed in cultures showing copy numbers above 95% LOD in qPCR testing at week 3 (*n*=2/3 plasma cultures, 66.7%; *n*=3/3 CSF cultures, 100.0%) (Table 1). Accordingly, copy numbers of culture-positive samples were significantly higher than those of qPCR-positive culture-negative samples (median 3.6×10^6^ vs 4.8×10^2^ copies per millilitre, *P*=0.001).

Further, we reactivated a subset of 20 frozen week 3 cultures (*n*=15 plasma and *n*=5 CSF) that had not shown viable spirochetes by microscopy at week 9. It included all qPCR-positive cultures (*n*=11 plasma and *n*=3 CSF) for which frozen samples were available, and randomly selected 6 qPCR-negative cultures (*n*=4 plasma and *n*=2 CSF) (Table S2). These cultures were incubated and monitored by microscopy for an additional 9 weeks (12 weeks of total incubation). None of the reactivated cultures showed copy numbers above the 95% LOD in qPCR testing for *B. burgdorferi* DNA, and none of them contained viable spirochetes when examined by microscopy.

## Discussion

The aim of this study was to optimize the protocol for cultivating *B. burgdorferi* from patient samples in a clinical setting, prioritizing the cultures more likely to become *B. burgdorferi* positive.

We obtained positive *B. burgdorferi* cultures for 15.6% of the LD patient samples, which is consistent with previously reported sensitivities for culturing *B. burgdorferi* from clinical samples (1.5–44%) [8,10,13, 26]. Earlier studies have indicated that the success of *B. burgdorferi* cultivation is mainly influenced by the volume of the patient sample inoculated into the culture medium [4,7]. In paediatric populations, the volumes of plasma and CSF samples available for research are particularly limited, as most of these specimens are prioritized for routine diagnostic testing. In addition, the success of culturing *B. burgdorferi* crucially depends on the culture conditions, such as a strict temperature window, microaerophilic atmosphere and the use of freshly prepared culture media. *B. burgdorferi* cultures are typically incubated at 33–34 °C [4,8], but comparable growth has also been observed between 33 and 37 °C [5,6]. In our study, we incubated the cultures at 37 °C in a microaerophilic atmosphere to replicate and maintain the temperature found in the human host. We used MKP medium, which is recommended for isolating *B. burgdorferi* from clinical samples [4,27, 28]. Finally, a laboratory strain was cultured under the same conditions as the control to demonstrate consistent and reliable growth throughout the entire culture period. Overall, our results demonstrate that the described procedure can be used to obtain positive *B. burgdorferi* cultures from as little as 1 ml of patient plasma or CSF sample.

Several qPCR assays have been reported for the detection of *B. burgdorferi* DNA, most frequently targeting *flaB*, *hbb*, *OspA*, *16S rRNA* or intergenic spacer *5S*–*23S rRNA* genes [23,29,31]. The qPCR assay used in this study, targeting the *flaB* gene, produced a consistently high amplification signal and detected *B. burgdorferi* DNA in all five culture-positive samples, as well as in laboratory strains. The use of NTCs and an internal extraction control confirmed the absence of contamination and successful DNA extraction. Further, the slopes and coefficients of determination (R-squared) of the generated standard curves were comparable to those described by Schwaiger *et al*. [23].

While the qPCR assay was able to detect *B. burgdorferi* genetic material in 22 cultures at week 3 of incubation, bacteria were cultivable only in 5 (22.3%) cases. It should be noted that all PCR-positive assays from culture-negative samples had much lower estimated copies of *B. burgdorferi* DNA than culture-positive samples. We hypothesize that low levels of *B. burgdorferi* DNA detectable in cultures are derived either from viable, non-dividing bacteria or from residual DNA from dead bacteria [32].

**Fig. 2.**
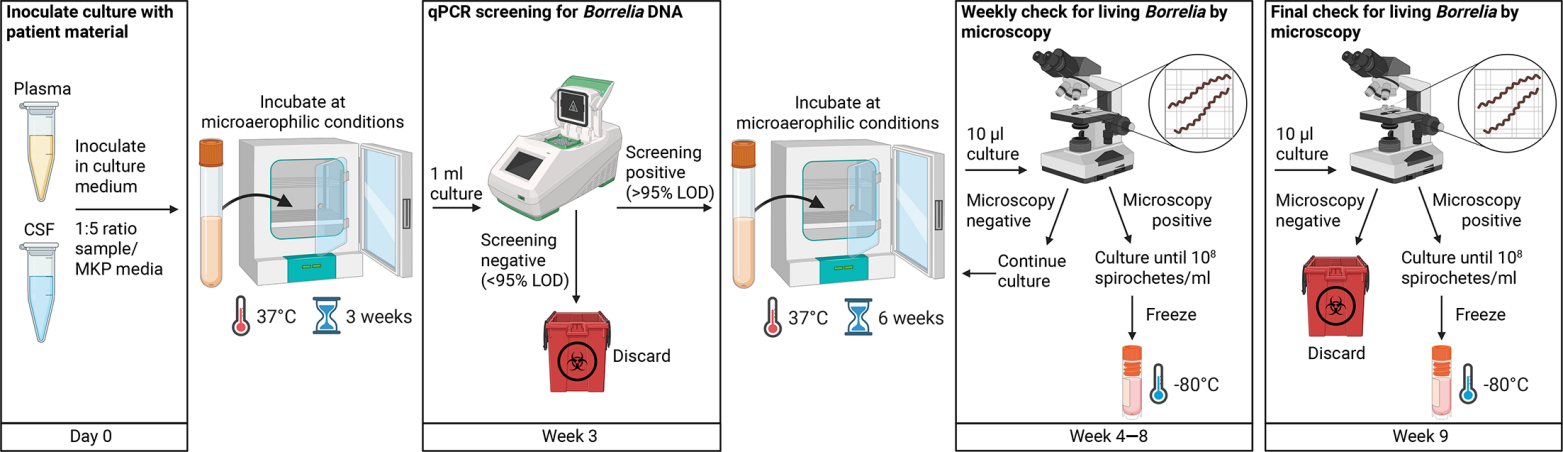
Scheme for streamlining *B. burgdorferi* cultivation. Modified process for the cultivation of *B. burgdorferi* out of clinical samples. Day 0: inoculate the clinical specimen into MKP medium at a ratio of 1:5 on the day of sampling and incubate for 3 weeks without disturbing it, under microaerophilic conditions at 37 °C. Week 3: test for *B. burgdorferi* DNA by qPCR after 3 weeks of incubation. Cultures that tested negative for *B. burgdorferi* DNA or showed an estimated copy number of target sequence below the qPCR assay’s 95% LOD can be discarded. Weeks 4–8: cultures tested positive with an estimated copy number of target template higher than the 95% LOD at week 3 should be incubated up to 9 weeks with weekly checking for living bacteria by phase-contrast microscopy. Cultures containing viable spirochetes should be washed and frozen in 10% glycerol at −80 °C when reaching a bacterial concentration of 1x10^8^ bacteria per 1 millilitre. Week 9: negative cultures will be disposed of after 9 weeks of cultivation. Abbreviation: CSF, cerebrospinal fluid; LOD, limit of detection; MKP, modified Kelly-Pettenkofer.

We found no viable spirochetes under the microscope after 9 and 12 weeks of incubation, when estimated *B. burgdorferi* DNA copies were below the 95% LOD at week 3. In contrast, in cultures with a copy number above the 95% LOD, viable spirochetes could be detected by microscopy in 66.7% (*n*=2/3) of plasma and 100% (*n*=3/3) of CSF cultures. These findings suggest that qPCR could be a useful tool for screening early cultures for *B. burgdorferi* DNA and identifying those with a high likelihood of harbouring cultivable bacteria. This approach could help streamline the culture process and reduce the overall workload (Fig. 2). In brief, clinical samples (≥1 ml) are inoculated 1 : 5 in MKP medium and incubated under microaerophilic conditions at 37 °C for 3 weeks. qPCR is then performed on 1 ml of culture. Samples with an amount of target template above the assay’s 95% LOD are cultivated for a further 6 weeks and monitored weekly by microscopy. Cultures with a lower number of target templates are discarded. If viable spirochetes are observed, cultures are frozen at 10⁸ bacteria per millilitre. qPCR-positive but microscopy-negative cultures are discarded after 9 weeks.

Limitations of the study include its relatively small sample size and a homogenous patient cohort. Moreover, the absence of healthy control patients prevented comparison with the possibility of incidental detection of *B. burgdorferi* bacteria or DNA in the blood of children without suspected infection. These factors may limit the external validity of the findings and should be taken into account when interpreting the results. Accordingly, the utility of the proposed qPCR-based screening assay should be assessed in a larger and more diverse population, including adult patients, to confirm its broader applicability and performance across different demographic and clinical settings.

In conclusion, while the *in vitro* cultivation of *B. burgdorferi* poses significant challenges, it remains feasible under appropriate conditions. Further investigation of clinical *B. burgdorferi* isolates in larger studies is essential to advance our understanding of the *B. burgdorferi* biology and LD pathogenesis. In this study, we demonstrated that qPCR is an effective tool for detecting and quantifying *B. burgdorferi* DNA in liquid cultures, thereby facilitating cultivation and maintenance of the cultures. However, the absence of standardized international quantification protocols for *B. burgdorferi* impedes the development of standardized calibration curves and LOD cut-off values. The establishment of such standards would enhance the comparability of qPCR data across studies and would improve the reproducibility of results.

## Supplementary material

10.1099/jmm.0.002123 Uncited Fig. S1.

## Abbreviations

Cq: quantification cycle
CSF: cerebrospinal fluid
EM: erythema migrans
*flaB*: *flagellin B*
LA: Lyme arthritis
LD: Lyme disease
LNB: Lyme neuroborreliosis
LOD: limit of detection
MKP: modified Kelly-Pettenkofer
NTCs: no template controls
PhHV: phocine herpesvirus
qPCR: quantitative PCR
RT: room temperature
