# Supplementary material for: Streamlining Borrelia burgdorferi cultivation using quantitative PCR screening
Source: J Med Microbiol. 2026 Feb 6;75(2):002123. doi: 10.1099/jmm.0.002123 (PMC12881930; doi:10.1099/jmm.0.002123)
Supplement: Uncited Fig. S1. [file jmm-75-02123-s001.pdf]

Supplementary appendix

**Table S1:**	Primers and probes used for duplex qPCR reaction	Page 2
**Table S2:**	Results of *Borrelia burgdorferi*-specific qPCR testing after 12 weeks and weekly microscopy over 12 weeks of cultivation of 15 plasma and 5 CSF cultures	Page 3
**Figure S1:**	Establishment of estimated 95% limit of detection (95% LOD) of qPCR assay for the detection of *Borrelia burgdorferi* DNA in clinical cultures	Page 4
**Protocol S1:**	Recipe for Modified Kelly-Pettenkofer (MKP) medium	Page 5–6

Supplementary Table S1. Primers and probes used for duplex qPCR reaction.

**Primers/probe**						
**Target**	**Sequence (5' → 3')**		**5'-Dye and 3'-Quencher**		**Concentration in qPCR reaction**	**Reference**
flaB	Forward:	AGCAAATTTAGGTGCTTTCCAA	Dye:	FAM	0.9 nM	Schwaiger et al. (2001)[b]
	Reverse:	GCAATCATTGCCATTGCAGA	Quencher:	BHQ-1	0.9 nM	
	Probe:	TGCTACAACCTCATCTGTCATTGTAGCATCTTTTATTTG			0.25 nM	
**PhHV**	Forward:	GGGCGAATCACAGATTGAATC	Dye:	HEX	0.9 nM	Van Doornum et al. (2003)[c]
	Reverse:	GCGGTTCCAAACGTACCAA	Quencher:	BHQ-2	0.9 nM	
	Probe:	TTTTATGTGTCCGCCACCATCTGGATC			0.25 nM	

***flaB* plasmid standard**	
	**Sequence (5' → 3')[a]**
	GCAATCATTGCCATTGCAGATTGTGTTAAAATACTATTAGTTGTAGCTGCTACAACCTCATCTGTCATTGTAGCATCTTTTATTTGA GCATAAGATGCTTTTAGGTTTTCAATAGCATACTCAGTACTATCCTTTATAGACTCAAGTCTATTTTGGAAAGCACCTAAATTTGCT

[a] *flaB* gene sequence from *Borrelia burgdorferi* strain B31 (NCBI RefSeq assembly GCF_000008685.2).
[b] Schwaiger M, Peter O, Cassinotti P. Routine diagnosis of *Borrelia burgdorferi* (sensu lato) infections using a real-time PCR assay. Clin Microbiol Infect. 2001;7(9):461-469.
[c] van Doornum GJ, Guldemeester J, Osterhaus AD, Niesters HG. Diagnosing herpesvirus infections by real-time amplification and rapid culture. J Clin Microbiol. 2003;41(2):576-580.

Abbreviation: *flaB*, *Borrelia burgdorferi flagellin B* gene; PhHV, phocine herpes virus 1.

Supplementary Table S2. Results of *Borrelia burgdorferi*-specific qPCR testing after 12 weeks and weekly microscopy over 12 weeks of cultivation of 15 plasma and 5 CSF cultures.

		Plasma				CSF			
		qPCR 12 weeks		Microscopy 12 weeks		qPCR 12 weeks		Microscopy 12 weeks	
Patient no.	Diagnosis	Copies/mL [a,b]	Interpretation[c]	Spirochetes first detected (day)[d]	Interpretation	Copies/mL [a,b]	Interpretation[c]	Spirochetes first detected (day)[d]	Interpretation
4	LNB	1372	Positive (<LOD)	NA	Negative	3236	Positive (<LOD)	NA	Negative
8	LNB	NS	Negative	NA	Negative	616	Positive (<LOD)	NA	Negative
5	LNB	4091	Positive (<LOD)	NA	Negative	176	Positive (<LOD)	NA	Negative
7	LNB	NS	Negative	NA	Negative	46	Positive (<LOD)	NA	Negative
11	LNB	NS	Negative	NA	Negative	NS	Negative	NA	Negative
9	LNB	306	Positive (<LOD)	NA	Negative				
10	LNB	NS	Negative	NA	Negative				
1	LNB	NS	Negative	NA	Negative				
13	LNB	NS	Negative	NA	Negative				
15	EM	582	Positive (<LOD)	NA	Negative				
16	EM	NS	Negative	NA	Negative				
17	EM	NS	Negative	NA	Negative				
22	EM	NS	Negative	NA	Negative				
28	LA	NS	Negative	NA	Negative				
31	LA	NS	Negative	NA	Negative				

[a] Estimated copies of target template in 1 mL of culture.
[b] NS (no signal) – no amplification signal detected by qPCR above baseline threshold.
[c] Interpretation of qPCR testing: negative – no *B. burgdorferi* DNA detected; positive (<LOD) – *B. burgdorferi* DNA detected, but signal is below 95% LOD; positive (>LOD) – *B. burgdorferi* DNA detected and signal above 95% LOD. 95% LOD: 6.38 x 10^3 copies/mL or 3.80 log(10) of quantity/mL (**Supplementary Figure S1**).
[d] Day after incubation start when living spirochetes were first detected by phase contrast microscopy, 400x magnification.

Abbreviation: CSF, cerebrospinal fluid; EM, erythema migrans; LA, Lyme arthritis; LNB, Lyme neuroborreliosis; LOD, limit of detection; NA, not available; NS, no signal.

Supplementary Figure S1. Establishment of estimated 95% limit of detection (95% LOD) of qPCR assay for the detection of *Borrelia burgdorferi* DNA in clinical cultures.

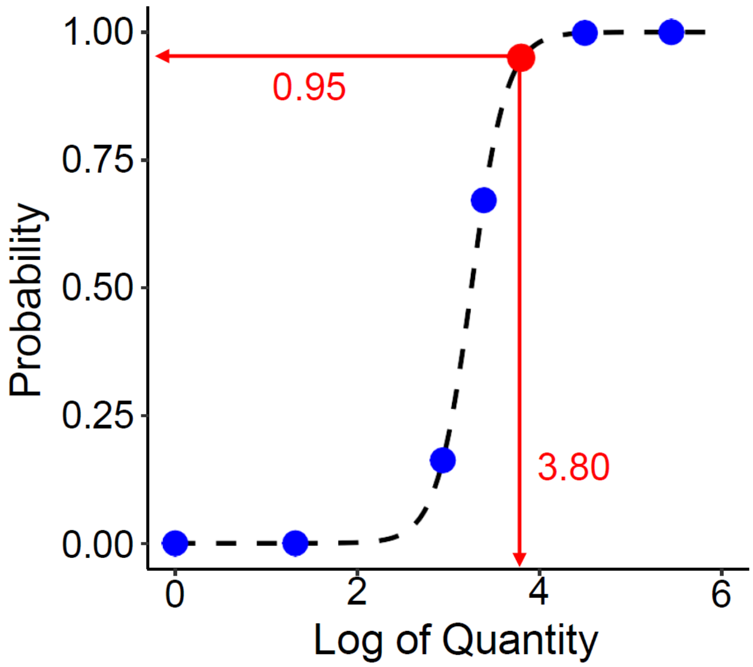

Graphical representation of the generalized linear model with logit link function. It visualizes the probability of a positive qPCR result in all tested replicates as a function of the log(10) of quantity of the target template per 1 mL of bacterial culture (dashed line). Blue dots show the data points used to build the model based on the serial dilutions (6 x 1:10 dilutions, ranging from 4.19 x 10^5 to 4.19 x 10^0 *B. burgdorferi* DNA copies/mL) of well-growing *B. burgdorferi* culture. Red dot shows the estimated lowest amount of target sequence per 1 mL of culture that can be detected by the qPCR assay in 95% of the replicates (95% LOD, 3.80 log(10) of quantity corresponding to 6.38 x 10^3 copies).

Supplementary Protocol S1. Recipe for Modified Kelly-Pettenkofer (MKP) medium.

Part 1: MKP (Modified Kelly-Pettenkofer) basic medium

Material and composition

**Table 1. Composition of 1 L of basic MKP medium**	
**Constituents**	**Amount (/L)**
CMRL-1066 (10x without glutamine)	100 mL (if liquid)/9.7 g/L if powder
Neopeptone	3 g
Hepes	6 g
Citric acid	0.7 g
Glucose	3 g
Pyruvic acid	0.8 g
N-acetylglucoseamine	0.4 g
Sodium bicarbonate	2 g

- Distilled water (ddH2O)
- NaHO

Storage

Basic medium can be aliquoted (i.e., 50 mL) and stored at -20°C for up to 3 months.

Laboratory procedure

Work in sterile manner on bench.

1) Weigh and dissolve all powdered components listed in Table 1 in ddH2O. Use ¾ of the final volume (of basic medium) of ddH2O (i.e., for 1 L of basic medium use 750 mL of ddH2O for dissolving). Stir until complete dissolving (takes around 1 h).
2) After complete dissolving, adjust the pH with NaHO to 7.6 and the volume to 1000 mL (use ddH2O). Sterilize by filtration (0.22 µm filter).
3) Aliquot and freeze basic medium.

Part 2: MKP (Modified Kelly-Pettenkofer) complete medium

Material and composition

**Table 2. Composition of MKP (Modified Kelly-Pettenkofer) complete medium**	
**Constituents**	**Amount (mL)**
Basic medium	500
7% gelatin (in H2O) (freshly autoclaved but not hot)	100
Rabbit serum	36
35% BSA	17.5

Storage

Keep MKP complete medium at +4°C for no more than 1 month. Do not freeze.

Laboratory procedure

Work in sterile biological safety cabinet.

1) Mix all components listed in Table 2 under sterile conditions.
2) Sterilize the gelatin by autoclaving and handle in a sterile manner before adding to the mixture.
3) Use a sterile filtered serum.
4) Aliquot MKP in appropriate volumes (i.e., 10 mL)
5) Put one aliquot at 33-37°C for several day. Then check under darkfield or phase contrast microscope for the absence of contamination.

NB! The complete medium can be additionally sterilized by filtration (0.22 µm filter).
